# The uptake of family screening in hypertrophic cardiomyopathy and an online video intervention to facilitate family communication

**DOI:** 10.1002/mgg3.940

**Published:** 2019-09-03

**Authors:** Stephanie Harris, Allison L. Cirino, Christina W. Carr, Hiwot M. Tafessu, Siddharth Parmar, Jeffrey O. Greenberg, Lara E. Szent‐Gyorgyi, Roya Ghazinouri, Michelle G. Glowny, Kara McNeil, Efthalia F. Kaynor, Catherine Neumann, Christine E. Seidman, Calum A. MacRae, Carolyn Y. Ho, Neal K. Lakdawala

**Affiliations:** ^1^ Cardiovascular Division Brigham and Women’s Hospital Boston Massachusetts; ^2^ Department of Medicine Brigham and Women’s Hospital Boston Massachusetts; ^3^ Present address: Cardiology Division Massachusetts General Hospital 55 Fruit Street Boston Massachusetts; ^4^ Present address: Herbert Wertheim College of Medicine Florida International University 11200 SW 8th Street Miami Florida; ^5^ Present address: Fox Chase Cancer Center 333 Cottman Avenue Philadelphia Pennsylvania

**Keywords:** cascade screening, family communication, genetic testing, hypertrophic cardiomyopathy, uptake

## Abstract

**Background:**

Individuals with hypertrophic cardiomyopathy (HCM), even when asymptomatic, are at‐risk for sudden cardiac death and stroke from arrhythmias, making it imperative to identify individuals affected by this familial disorder. Consensus guidelines recommend that first‐degree relatives (FDRs) of a person with HCM undergo serial cardiovascular evaluations.

**Methods:**

We determined the uptake of family screening in patients with HCM and developed an online video intervention to facilitate family communication and screening. Family screening and genetic testing data were collected through a prospective quality improvement initiative, a standardized clinical assessment and management plan (SCAMP), utilized in an established cardiovascular genetics clinic. Patients were prescribed an online video if screening of their FDRs was incomplete and a pilot study on video utilization and family communication was conducted.

**Results:**

Two‐hundred and sixteen probands with HCM were enrolled in SCAMP Phase I and 190 were enrolled in SCAMP Phase II. In both phases, probands reported that 51% of FDRs had been screened (382/749 in Phase I, 258/504 in Phase II). Twenty patients participated in a pilot study on video utilization and family communication. Nine participants reported watching the video and six participants reported sharing the video with relatives; however only one participant reported sharing the video with relatives who were not yet aware of the diagnosis of HCM in the family.

**Conclusion:**

Despite care in a specialized cardiovascular genetics clinic, approximately one half of FDRs of patients with HCM remained unscreened. Online interventions and videos may serve as supplemental tools for patients communicating genetic risk information to relatives.

## 1 INTRODUCTION

Hypertrophic cardiomyopathy (HCM) is characterized by left ventricular hypertrophy (LVH) caused primarily by variation in sarcomere genes and is the most common genetic cardiomyopathy (Garfinkel, Seidman, & Seidman, 2018). Individuals with HCM, even when asymptomatic, are at‐risk for sudden cardiac death and stroke from ventricular and atrial arrhythmias, respectively, making it imperative to identify individuals with disease. Screening can avert these complications through placement of implantable cardioverter‐defibrillators (ICDs) or initiation of anticoagulation where indicated.

Sarcomere variants are transmitted in an autosomal dominant fashion with age‐dependent penetrance and variable clinical expression. Accordingly, consensus guidelines recommend that first‐degree relatives (FDRs) of a person with HCM undergo serial cardiovascular evaluations including electrocardiogram (ECG) and echocardiogram (Gersh et al., 2011). In families where a disease‐causing variant has been identified, cascade genetic testing allows at‐risk relatives to be definitively identified and those without the familial variant to be excused from cardiovascular evaluations. In families where genetic testing is not performed or is uninformative, the default strategy of serial cardiovascular evaluations for FDRs is employed.

Genetic risk information is generally disseminated through a family by the patient. Due to ethical and privacy concerns, health‐care providers typically do not contact patients' relatives directly to disclose risk information (Dugan et al., 2003; Falk, Dugan, O’Riordan, Matthews, & Robin, 2003; Forrest, Delatycki, Curnow, Skene, & Aitken, 2010). However, this approach may lead to incomplete uptake of family screening; previous research in patients with HCM, dilated cardiomyopathy (DCM), and long QT syndrome (LQTS) has shown that the uptake of family screening (cardiovascular evaluations or genetic testing) is approximately 40%–60% (Burns, Mcgaughran, Davis, Semsarian, & Ingles, 2016; Christiaans, Birnie, Bonsel, Wilde, & van Langen, 2008; Miller, Wang, & Ware, 2013).

A variety of factors may influence family communication of genetic risk and the subsequent uptake of screening, including demographic factors (gender, socioeconomic status), family dynamics (emotional and geographical closeness, feelings of responsibility toward relatives), education or genetic literacy, psychosocial factors (depression, anxiety, guilt), and family history or clinical characteristics (Batte et al., 2015; Burns et al., 2016; Chivers Seymour, Addington‐Hall, Lucassen, & Foster, 2010; Sharaf, Myer, Stave, Diamond, & Ladabaum, 2013; Wiseman, Dancyger, & Michie, 2010). Although much of this research has been conducted in families with inherited cancer syndromes, a few studies have investigated the factors influencing communication and the uptake of screening in families with inherited cardiovascular disease. In a study of patients recruited through a patient advocacy group for HCM, female gender and understanding of autosomal dominant inheritance emerged as important factors influencing family communication (Batte et al., 2015). Additionally, socioeconomic status, anxiety, and depression have been suggested to influence family communication and the uptake of genetic testing in LQTS (Burns et al., 2016).

Many centers have adopted the practice of providing written information or a “family letter” to patients to share with their relatives (Forrest et al., 2010), and research suggests that family letters may improve screening uptake (Van Der Roest, Pennings, Bakker, Van Den Berg, & Van Tintelen, 2009). However, there are disadvantages to this approach and alternative strategies, including online tools, are needed to facilitate family communication and screening (Dheensa, Lucassen, & Fenwick, 2018; Sturm, 2016). There are limited studies that assess interventions aimed to improve family communication and the uptake of family screening, and results are mixed. In two separate randomized‐controlled trials, a counseling intervention and communication skills‐building intervention did not demonstrate a significant impact on screening uptake (Hodgson et al., 2016; Montgomery et al., 2014). Alternatively, another study found that an intervention focused on providing additional genetic counseling support led to a significant increase in the uptake of genetic services by at‐risk relatives (Forrest, Burke, Bacic, & Amor, 2008). The aim of our study was to determine the uptake of family screening in a cohort of patients with HCM and to complete a pilot study assessing a novel online video intervention to facilitate family communication and screening.

## 2 METHODS

### 2.1 Editorial policies and ethical considerations

This study was approved by the Partners Human Research Committee (IRB). All procedures involving human participants were in accordance with the 1964 Helsinki declaration and its later amendments or comparable ethical standards. Informed consent was obtained from all participants prior to inclusion the study.

### 2.2 Standardized clinical assessment and management plan initiative

Family screening and genetic testing data were collected through a prospective quality improvement (QI) initiative, a standardized clinical assessment and management plan (SCAMP), that has been reported as an effective tool for improving care practices among patients with rare diseases (Farias et al., 2013). The goal of the SCAMP was to improve the care provided to patients with HCM by implementing a practice algorithm and studying deviations from the algorithm. One specific focus of the SCAMP was implementing the recommendation that FDRs of a proband with HCM undergo cardiovascular evaluation or cascade genetic testing when appropriate.

The SCAMP practice algorithm was designed by a team of cardiologists, genetic counselors, and statisticians. New and returning patients were eligible for enrollment in the SCAMP if they had a clinical diagnosis of HCM and were receiving longitudinal clinical care in the Brigham and Women's Cardiovascular Genetics Clinic. Patients with hypertensive LVH, Fabry disease, amyloidosis, and other systemic conditions associated with LVH were excluded. Family screening data was based on report from the affected relative enrolled in the SCAMP (proband). First‐degree at‐risk relatives were considered unscreened if they had not pursued cardiovascular evaluation (including electrocardiogram and echocardiogram) or cascade genetic testing, when appropriate; or if they had previously pursued cardiovascular evaluation but their evaluation was outdated per consensus guidelines (Gersh et al., 2011). Implementation of the practice algorithm and data collection occurred via paper forms (Appendices 1 and 2). Eligible patients were identified by administrative staff and/or providers; providers completed the data collection form at the time of the outpatient encounter; a data coordinator distributed and collected data forms and reminded providers to complete outstanding data forms, achieving a data form completion rate of 85%.

SCAMPs are modifiable practice algorithms that allow for continuous improvement and standardization of clinical practice. There were two phases of the HCM SCAMP: Phase I took place from July 2013 to July 2014; Phase II took place from September 2015 to July 2016. The practice algorithm underwent revision in between the two phases; one of the goals of revision in Phase II was to refine and expand data collection on the uptake of family evaluations. Additionally, the Phase II SCAMP algorithm included the new recommendation that providers prescribe an educational video (described below) to any proband where the uptake of evaluations in FDRs was incomplete.

### 2.3 Educational video

Online educational videos were created in the Brigham and Women's Cardiovascular Genetics Clinic in April 2015. Two videos were created, namely one video was intended for families with a disease‐causing variant where cascade genetic testing was available to at‐risk relatives; the second video was intended for families where genetic testing had not been performed or was uninformative (Appendix 3). The videos are approximately 5‐min long and feature two genetic counselors reviewing the diagnosis of HCM, autosomal dominant inheritance, the recommendations for cardiovascular evaluations for FDRs, and the availability of genetic testing. Patients in the Cardiovascular Genetics Clinic were prescribed a video if screening of their at‐risk relatives was incomplete. Videos were prescribed by the provider during or in follow‐up after a patient encounter via an emailed hyperlink or paper handout containing the video URL. The video was viewable in all web browsers.

A pilot study investigating patients’ utilization of the video and family communication preferences was conducted from August 2017 to November 2017. Patients were eligible for participation in the study if they met the following criteria: 18 years of age or older, had a clinical diagnosis of HCM, were prescribed a video by their provider, and were English‐speaking. Patients were not required to be enrolled in the SCAMP to be eligible for the study (e.g., patients not receiving longitudinal care at our center would have been ineligible for the SCAMP but may have been provided the video and eligible to participate in the pilot study). Eligible patients were recruited at the time of clinic visits or by telephone. Surveys were administered by a research assistant or genetic counselor in person or over the telephone; alternatively, participants could complete the survey online via a link through their email. Participants who opted to complete the survey online were sent a reminder 2 weeks later if the survey had not yet been completed.

Participants completed a non‐anonymous survey consisting of multiple‐choice questions, Likert scales, and free response questions (Appendices 4 and 5). The survey questions pertained to the uptake of family screening, participant perception of the video, video sharing practices, the impact of the video on the screening practices of at‐risk relatives, and family communication preferences. The survey was managed and distributed through RedCap.

### 2.4 Data analysis

SCAMP data were analyzed by the Institute for Relevant Clinical Data Analytics, Inc. Descriptive statistics were generated to characterize the demographic and genetic testing data for the SCAMP cohort. *T* tests were completed to assess the differences in uptake of family screening based on genetic test result and the uptake of family screening based on gender. Linear regression analyses were used to assess the uptake of family screening based on the number of visits a patient had in the Cardiovascular Genetics Clinic, as well the uptake of family screening based on proband age. Chi‐squared analysis or Fisher's exact test was used to assess differences between genetic testing groups for reasons family members were not evaluated. Survey data were analyzed in Excel.

## 3 RESULTS

### 3.1 Uptake of family screening

Two‐hundred and sixteen patients with HCM were enrolled in SCAMP Phase I and 190 patients were enrolled in SCAMP Phase II. Demographic data are summarized in Table 1. Genetic testing data were not recorded for 23 subjects in SCAMP Phase I and 49 subjects in Phase II. In SCAMP Phase I genetic testing was performed in 112 of 193 (58%) probands (66 probands had a positive result, 11 had variant of uncertain significance [VUS], and 35 had a negative result) and 81 of 193 (42%) probands did not pursue genetic testing. In SCAMP Phase II genetic testing was performed in 96 of 141 (68%) probands (45 had a positive result, 11 had a VUS, and 24 had a negative result, and results were unavailable for 16 patients); 45 of 141 (32%) probands did not pursue genetic testing. In Phase I, the 193 probands had 749 FDRs, of which 382 (51%) had been screened. In phase II, the 141 probands had 504 FDRs, of which 258 (51%) had been screened.

**Table 1:** Cohort demographics and uptake of proband genetic testing in standardized clinical assessment and management plan phase I and phase II

	Phase I	Phase II
Probands (*n*)	216	190
Age (mean ± SD)	52.7 ± 15.6	54 ± 15.4
Gender		
Female	96	75
Male	120	115
Race		
White	186	159
Black	10	10
Asian	6	7
Hispanic	3	1
Other	3	4
Not reported	8	9
Commercial insurance	149	136
Genetic testing		
Genetic testing performed	112	96
Positive	66	45
VUS	11	11
Negative	35	24
Result unavailable	—	16
Genetic testing not performed	81	45
Total	193	141

Abbreviation: VUS, variant of uncertain significance.

**Figure 1:**
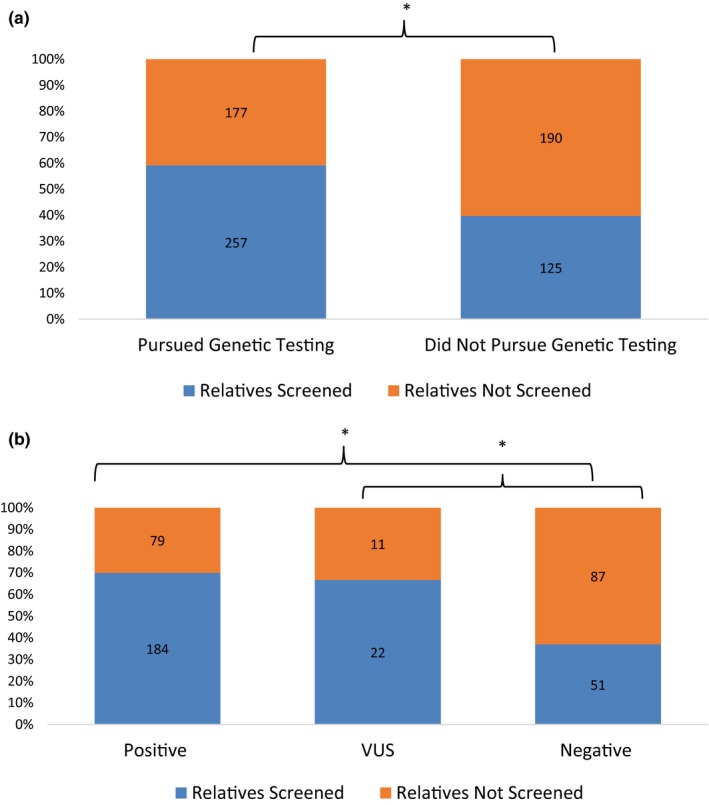
(a) First‐degree relatives (FDRs) of probands who pursued genetic testing were significantly more likely to have been evaluated than the FDRs of probands who declined genetic testing. (b) The FDRs of probands with a positive or variant of uncertain significance result were significantly more likely to have been evaluated than the FDRs of a proband with a negative result. *p < .05

The influence of proband genetic testing on the uptake of family screening was assessed in Phase I. In the 112 probands who pursued genetic testing, 257 of the 434 (59%) of their FDRs had been appropriately screened. In comparison, in the 81 probands who did not pursue genetic testing, only 125 of 315 (40%) of their FDRs had been screened (*p* < .05) (Figure 1a). In the probands who pursued genetic testing, 184 of 263 (70%) and 22 of 33 (67%) of the FDRs of probands with a positive result or VUS, respectively, were screened; as compared to only 51 of 138 (37%) of FDRs of probands with a negative result (positive vs. negative, VUS vs. negative *p* < .05) (Figure 1b).

In Phase II, other factors that may influence the uptake of family screening were investigated. There was no significant difference between proband gender and the mean uptake of family screening (*p* = .89). Additionally, there was no significant association between proband age and uptake of family screening (*N* = 141, parameter estimate = −0.003, 95% CI −0.008 to 0.001, *p* = .165). However, there was a significant, positive linear association between the number of visits a patient had in the cardiovascular genetics clinic and the uptake of family screening. (*N* = 141, parameter estimate = 0.013, 95% CI 0.002–0.024, *p* = .0217).

The reasons relatives did not pursue screening, as reported by the proband, were also assessed in Phase II and dichotomized by whether the proband had undergone genetic testing in Table 2. Eighty‐eight of 141 (62%) probands had less than 100% uptake of family screening. Of these probands, 61 of 88 (69%) reported their relatives were aware of the recommendation for screening but were not interested in being evaluated. Only 6 of 88 (7%) of probands reported that they were aware of the recommendations for family screening but had not yet communicated this information to their relatives. Four of 88 (5%) probands reported they were unaware of the recommendation for family screening at the time of their appointment.

**Table 2:** Reasons relatives were not screened in standardized clinical assessment and management plan phase II

	Pursued genetic testing	Did not pursue genetic testing	Total
Probands (*n*)	96	45	141
Probands with <100% of relatives screened	55 (57%)	33 (73%)	88 (62%)
Reasons relatives not screened			
Probands (*n*)	55	33	88
Family aware but not interested	45 (82%)*	16 (48%)*	61 (69%)
Proband aware but has not communicated with family	4 (7%)	2 (6%)	6 (7%)
Proband unaware of family screening recommendation	1 (2%)	3 (3%)	4 (5%)
New diagnosis in proband	N/A	12 (36%)	12 (14%)
Other	7 (13%)	1 (3%)	8 (9%)

* *p* < .05.

### 3.2 Video utilization

At least 82 patients were prescribed an educational video and eligible to participate in a pilot study. Forty‐three patients were approached and 20 of 43 (46.5%) participated in the survey, four of 43 (9.3%) declined participation, and 19 of 43 (44.2%) patients did not respond. Nine of 20 participants reported watching the video, three of 20 did not watch the video, and eight of 20 were unsure if they had watched it. Participants who watched the video were asked to complete 5‐point Likert scales to rate whether the video improved their understanding of HCM inheritance and family screening recommendations. Approximately half of the participants reported the video did not increase their understanding of risk to family members (1/7), or that they did not learn new information, but it was a helpful review (3/7); whereas two participants reported it increased their understanding “a little” and 1 reported it increased understanding “a lot.” Similarly, over half of participants reported the video did not increase their understanding of family screening recommendations (2/7) or that they did not learn new information, but it was a helpful review (3/7); whereas two participants reported they learned “a little.” Not all participants completed the 5‐point Likert scales.

**Figure 2:**
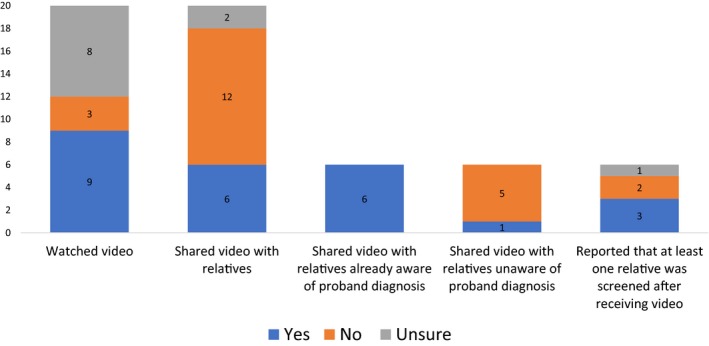
Approximately half of patients reported watching the video and six patients shared the video with relatives. Patients rarely shared the video with any relatives who were not already aware of their diagnosis. Half of the patients who shared the video reported at least one relative was evaluated after receiving the video

Six of 20 participants reported sharing the video with relatives; 12 of 20 did not share the video, and 2 of 20 were unsure if they had shared the video (Figure 2). All participants who shared the video reported sharing it with relatives who were already aware of the diagnosis of HCM in the family. Only one participant reported sharing the video with relatives who were not already aware of the diagnosis of HCM in the family. Three of six participants reported that at least one of their relatives had been screened after receiving the video; two of six did not think their relatives had been screened after receiving the video and one of the six patients was unsure.

**Figure 3:**
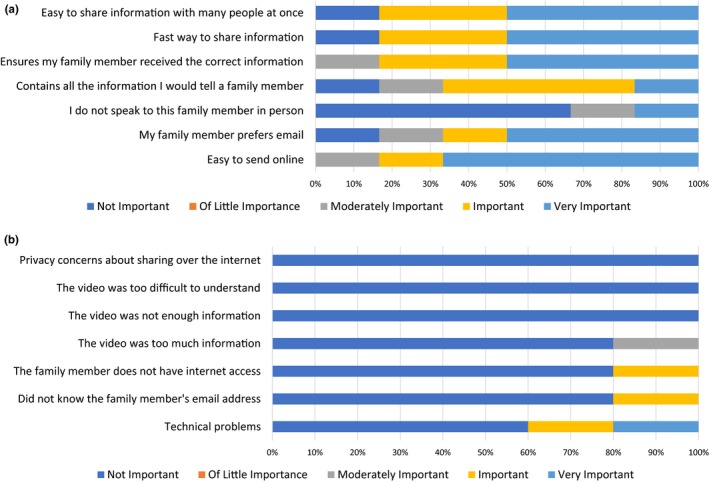
(a) Probands reported that the most important perceived motivators for sharing the video were its content and convenience. (b) Probands report few perceived barriers to sharing the video

Participants completed a series of 5‐point Likert scales assessing motivators and barriers to sharing the video with relatives (Figure 3). The majority of participants rated the following reasons as “important” or “very important” reasons for sharing the video: it was easy to send online (5/6), their relative prefers email (4/6), it contains all the information they would want to tell their relative (4/6), it ensures they are sharing the correct information (5/6), it is a fast way to share information (5/6), and it is an easy way to share information with multiple people at once (5/6). Most participants (4/6) reported that sharing the video to communicate with relatives with whom they do not usually speak to was “not important.” Most participants reported the following potential barriers to sharing the video were not important factors: technical problems (3/5), not knowing their relative's email address (4/5), having relatives without internet access (4/5), the video was too much information (4/5), the video did not provide enough information (5/5), the video was too difficult to understand (5/5), or privacy concerns about sharing the video over the internet (5/5).

Participants were asked about their family communication preferences. Patients endorsed the following educational materials as potentially helpful when sharing their diagnosis with family members: an educational video (11/19), a short brochure (5/19), a personalized letter (4/19), a detailed packet of information (2/19), other (5/19), none of these (4/19). Eight of nineteen participants were interested in receiving additional guidance from their health‐care provider to help communicate their diagnosis. Participant preferences were varied when hypothetically asked about having their health‐care provider contact their family members directly, but with their permission. Three participants were willing to have their provider contact all at‐risk relatives versus some at‐risk relatives (1/19), all at‐risk relatives after they had a chance to speak with them (7/19), some at‐risk relatives after they had a chance to speak with them (4/19), and none of their relatives (4/19).

## 4 DISCUSSION

The aim of our study was to systematically assess the uptake of family screening in a cohort of patients with HCM and to conduct a pilot study of an online video intervention to facilitate family communication and screening. Data from a SCAMP study determined that approximately half of the FDRs in our cohort of patients with HCM remained unscreened. This is consistent with the uptake of screening reported in other cohorts with inherited cardiomyopathies, inherited arrhythmias, and inherited cancer syndromes (Burns et al., 2016; Christiaans et al., 2008; Miller et al., 2013; Sharaf et al., 2013), emphasizing that a significant portion of at‐risk relatives across a variety of medically actionable inherited conditions do not seek appropriate care.

In the cohort of patients enrolled in the SCAMP, proband genetic test results were significantly associated with the uptake of family screening. The FDRs of probands who had undergone genetic testing were significantly more likely to have been screened, as compared to the FDRs of probands who had not undergone genetic testing. Additionally, in the group of probands who pursued genetic testing, the FDRs of probands with a positive or VUS result were significantly more likely to have been screened as compared to the relatives of probands with a negative result. This is consistent with the results from a study of families with LQTS, where at‐risk relatives in families with a pathogenic variant were significantly more likely to be screened than in families without a positive result (Hanninen et al., 2015).

In our study, only a small minority of probands (7%) reported that they had not communicated genetic risk and screening recommendations to their family members, and this was similar for probands who did and did not pursue genetic testing. The clear majority of probands (69%) stated that their relatives were aware of the recommendation for screening but were simply uninterested. Taken together, these results suggest that most probands communicate genetic risk information and screening recommendations to their relatives, but many family members choose not to act on this information. However, a positive genetic test result in the proband may serve as a significant motivator for relatives to seek evaluation.

These results echo the findings of a study where researchers, with proband permission, directly contacted FDRs of probands who had undergone *BRCA1/2* genetic testing. In this study, only 31.5% of relatives reported intention to pursue genetic *counseling* and 35% reported intention to pursue genetic *testing*, and intention was higher for relatives of probands with informative test results. Additionally, 10% of relatives in the study reported that the proband told them the result but they could no longer remember it; and 14% of relatives found the information was very or somewhat difficult to understand (Daly, Montgomery, Bingler, & Ruth, 2016). This suggests that despite probands’ intentions to disclose genetic test results, at‐risk relatives may have difficulty understanding this information and translating it into meaningful action. To inform the development of interventions targeting at‐risk relatives, additional research is needed to better understand the decision‐making of relatives who are aware of their risk but opt not to pursue screening.

Our results did not find a significant difference in proband gender on the uptake of screening, whereas female gender has been shown in multiple studies to be associated with increased family communication and screening (Batte et al., 2015; Koehly et al., 2009; Sharaf et al., 2013). However, there was a positive, linear association between the number of visits a patient had in clinic and the uptake of family screening, suggesting that reminders and increased support may help facilitate family communication. This finding is consistent with the results of a randomized‐controlled trial investigating the impact of additional genetic counseling support, where subjects who received additional genetic counseling support up to 6 months after receiving a genetic diagnosis had a higher rate of family screening uptake (Forrest et al., 2008). This finding underscores that family communication is not a singular event, but rather a dynamic process and probands may benefit from support throughout the course of communicating genetic risk information to their relatives. In our pilot study, eight of 19 participants welcomed additional support from their health‐care provider in sharing their diagnosis with family members.

Due to ethical and privacy concerns, the standard that has emerged in most countries is for the proband to communicate with their relatives, rather than health‐care providers contacting at‐risk relatives directly (Forrest et al., 2010; Forrest, Delatycki, Skene, & Aitken, 2007). Probands may perceive this as a burden, and some struggle with how and when to tell their relatives this information, worry about their relatives’ reactions, are unsure of who to tell or what information to share, or do not have a way to communicate with relatives with whom they have lost contact (Smart, 2010; Vavolizza et al., 2015). An alternative approach is for health‐care providers to contact at‐risk relatives directly, although this strategy raises concerns regarding privacy, family dynamics, and the right of family members “not to know” (Newson & Humphries, 2005). Several studies investigating the impact of direct‐contact have demonstrated an increased uptake of genetic services and a general acceptance of this approach (Sermijn et al., 2016; Suthers, Armstrong, McCormack, & Trott, 2006), suggesting a role for direct‐contact in disseminating genetic risk information. In our pilot study, participants' preferences were varied when hypothetically asked about direct‐contact; however, most participants expressed acceptability of having their health‐care provider contact at least some family members directly. Additional research is needed to understand the acceptability and feasibility of direct‐contact in the United States (Sturm, 2016).

Acknowledging the large portion of relatives who remain unscreened, there has been a call to consider novel ways to facilitate family communication and promote screening (Burns, James, & Ingles, 2018; Dheensa et al., 2018; Sturm, 2016). We developed two educational, online videos for patients to share with their relatives and conducted a pilot study to investigate video utilization. Although participants endorsed many motivators and few barriers to sharing the video, less than half of participants shared the video with their relatives. Additionally, the participants who shared the video typically sent it to relatives who were already aware of the diagnosis in the family, indicating that patients may use the video as a supplementary tool when communicating genetic risk and screening recommendations. Only three of the 20 participants reported that at least one of their relatives was evaluated after receiving the video.

Although we utilized the robust methodology of the SCAMP for obtaining family screening data, a major limitation of our study is that family screening uptake was reported by the proband and not verified with at‐risk relatives or through medical records. One study demonstrated that 22% of the FDRs a proband reported having disclosed results to did not actually recall receiving this information (Daly et al., 2016). Therefore, it is possible the probands in our study over‐reported communicating genetic risk information to their relatives, or similarly, did not accurately report which relatives had been screened or the reasons their relatives remained unscreened. Additionally, the probands in our study were not necessarily the index case in their family and it is possible at‐risk relatives were previously alerted to their risk by another affected family member, impacting the uptake of screening and communication patterns reported in our cohort. Our study demonstrated a positive, linear association between the number of visits a patient had in clinic and the uptake of family screening; however, this may simply reflect that patients who had multiple follow‐up visits had more time to disseminate family risk information and their family members had more time to seek evaluation.

Another limitation is the small sample size for our pilot study on utilization of an educational video. A randomized‐controlled trial of the video was not conducted, rather the video was prescribed to patients with incomplete family screening and video recipients were retroactively contacted and asked to participate in a survey. Providers could deviate from the SCAMP recommendation to prescribe the video; therefore, not all probands with incomplete family screening necessarily received the video. Additionally, some patients who were not enrolled in the SCAMP were prescribed the video and considered eligible for the pilot study. Only 20 of the 82 eligible video recipients participated in the survey and eight of the 20 could not recall watching the video, suggesting there might be recall bias due to the time that elapsed between video prescription and survey administration. Additionally, our cohort consisted of patients who were predominately white and had private insurance. The utilization of online technologies may be different in other populations.

In conclusion, approximately half of relatives fail to pursue the potentially life‐saving screening that is recommended when HCM is diagnosed in a family, significantly diminishing the benefit of genetic diagnosis. Our research suggests that most probands share genetic risk information with their relatives, but many at‐risk relatives do not to act on this information. A positive genetic test result in the proband may serve as a significant motivator for relatives to seek evaluation. Participants identified multiple motivators and few barriers to sharing an educational video with their relatives to facilitate family communication; however, participants who shared the video typically sent it to relatives who were already aware of the diagnosis in the family. Therefore, online interventions may serve as helpful supplementary tools for disseminating genetic risk information in a family. As the use of genetic testing expands, it will be imperative to develop novel mechanisms for facilitating family communication and screening to achieve the greatest benefit of genetic diagnosis in a family.

## CONFLICT OF INTEREST

None declared.

## References

[mgg3940-bib-0001] Batte, B. , Sheldon, J. P. , Arscott, P. , Huismann, D. J. , Salberg, L. , Day, S. M. , & Yashar, B. M. (2015). Family communication in a population at risk for hypertrophic cardiomyopathy. Journal of Genetic Counseling, 24(2), 336–348. 10.1007/s10897-014-9774-8 25304619

[mgg3940-bib-0002] Burns, C. , James, C. , & Ingles, J. (2018). Communication of genetic information to families with inherited rhythm disorders. Heart Rhythm: the Official Journal of the Heart Rhythm Society, 15(5), 780–786. 10.1016/j.hrthm.2017.11.024 29175646

[mgg3940-bib-0003] Burns, C. , Mcgaughran, J. , Davis, A. , Semsarian, C. , & Ingles, J. (2016). Factors influencing uptake of familial long QT syndrome genetic testing. American Journal of Medical Genetics, Part A, 170(2), 418–425. 10.1002/ajmg.a.37455 26544151

[mgg3940-bib-0004] Chivers Seymour, K. , Addington‐Hall, J. , Lucassen, A. M. , & Foster, C. L. (2010). What facilitates or impedes family communication following genetic testing for cancer risk? A systematic review and meta‐synthesis of primary qualitative research. Journal of Genetic Counseling, 19(4), 330–342. 10.1007/s10897-010-9296-y 20379768

[mgg3940-bib-0005] Christiaans, I. , Birnie, E. , Bonsel, G. J. , Wilde, A. A. M. , & van Langen, I. M. (2008). Uptake of genetic counselling and predictive DNA testing in hypertrophic cardiomyopathy. European Journal of Human Genetics, 16(10), 1201–1207. 10.1038/ejhg.2008.92 18478037

[mgg3940-bib-0006] Daly, M. B. , Montgomery, S. , Bingler, R. , & Ruth, K. (2016). Communicating genetic test results within the family: Is it lost in translation? A survey of relatives in the randomized six‐step study. Familial Cancer, 15(4), 697–706. 10.1007/s10689-016-9889-1 26897130 PMC5010833

[mgg3940-bib-0007] Dheensa, S. , Lucassen, A. , & Fenwick, A. (2018). Limitations and pitfalls of using family letters to communicate genetic risk: A Qualitative Study with patients and healthcare professionals. Journal of Genetic Counseling, 27(3):689–701. 10.1007/s10897-017-0164-x 29094272 PMC5943374

[mgg3940-bib-0008] Dugan, R. B. , Wiesner, G. L. , Juengst, E. T. , O’Riordan, M. , Matthews, A. L. , & Robin, N. H. (2003). Duty to warn at‐risk relatives for genetic disease: Genetic counselors’ clinical experience. American Journal of Medical Genetics, 119C(1), 27–34. 10.1002/ajmg.c.10005 12704635

[mgg3940-bib-0009] Falk, M. J. , Dugan, R. B. , O’Riordan, M. A. , Matthews, A. L. , & Robin, N. H. (2003). Medical Geneticists’ duty to warn at‐risk relatives for genetic disease. American Journal of Medical Genetics Part A, 120A(3), 374–380. 10.1002/ajmg.a.20227 12838558

[mgg3940-bib-0010] Farias, M. , Jenkins, K. , Lock, J. , Rathod, R. , Newburger, J. , Bates, D. W. , … Greenberg, J. (2013). Standardized Clinical Assessment And Management Plans (SCAMPs) provide a better alternative to clinical practice guidelines. Health Affairs, 32(5), 911–920. 10.1377/hlthaff.2012.0667 23650325 PMC3990928

[mgg3940-bib-0011] Forrest, L. E. , Burke, J. , Bacic, S. , & Amor, D. J. (2008). Increased genetic counseling support improves communication of genetic information in families. Genetics in Medicine, 10(3), 167–172. 10.1097/GIM.0b013e318164540b 18344705

[mgg3940-bib-0012] Forrest, L. E. , Delatycki, M. B. , Curnow, L. , Skene, L. , & Aitken, M. (2010). Genetic health professionals and the communication of genetic information in families: Practice during and after a genetic consultation. American Journal of Medical Genetics Part A, 152(6), 1458–1466. 10.1002/ajmg.a.33385 20503321

[mgg3940-bib-0013] Forrest, L. E. , Delatycki, M. B. , Skene, L. , & Aitken, M. A. (2007). Communicating genetic information in families ‐ A review of guidelines and position papers. European Journal of Human Genetics, 15(6), 612–618. 10.1038/sj.ejhg.5201822 17392704

[mgg3940-bib-0014] Garfinkel, A. C. , Seidman, J. G. , & Seidman, C. E. (2018). Genetic pathogenesis of hypertrophic and dilated cardiomyopathy. Heart Failure Clinics, 14(2), 139–146. 10.1016/j.hfc.2017.12.004 29525643 PMC5851453

[mgg3940-bib-0015] Gersh, B. J. , Maron, B. J. , Bonow, R. O. , Dearani, J. A. , Fifer, M. A. , Link, M. S. , … Yancy, C. W. (2011). 2011 ACCF/AHA guideline for the diagnosis and treatment of hypertrophic cardiomyopathy. Journal of Thoracic and Cardiovascular Surgery, 142(6), e153–e203. 10.1016/j.jtcvs.2011.10.020 22093723

[mgg3940-bib-0016] Hanninen, M. , Klein, G. J. , Laksman, Z. , Conacher, S. S. , Skanes, A. C. , Yee, R. , … Krahn, A. D. (2015). Reduced uptake of family screening in genotype‐negative versus genotype‐positive long QT syndrome. Journal of Genetic Counseling, 24(4), 558–564. 10.1007/s10897-014-9776-6 25273952

[mgg3940-bib-0017] Hodgson, J. , Metcalfe, S. , Gaff, C. , Donath, S. , Delatycki, M. B. , Winship, I. , … Halliday, J. (2016). Outcomes of a randomised controlled trial of a complex genetic counselling intervention to improve family communication. European Journal of Human Genetics, 24(3), 356–360. 10.1038/ejhg.2015.122 26130486 PMC4755371

[mgg3940-bib-0018] Koehly, L. M. , Peters, J. A. , Kenen, R. , Hoskins, L. M. , Ersig, A. L. , Kuhn, N. R. , … Greene, M. H. (2009). Characteristics of health information gatherers, disseminators, and blockers within families at risk of hereditary cancer: Implications for family health communication interventions. American Journal of Public Health, 99(12), 2203–2209. 10.2105/AJPH.2008.154096 19833996 PMC2775786

[mgg3940-bib-0019] Miller, E. M. , Wang, Y. , & Ware, S. M. (2013). Uptake of cardiac screening and genetic testing among hypertrophic and dilated cardiomyopathy families. Journal of Genetic Counseling, 22(2), 258–267. 10.1007/s10897-012-9544-4 23054336

[mgg3940-bib-0020] Montgomery, S. V. , Barsevick, A. M. , Egleston, B. L. , Bingler, R. , Miller, S. M. , Malick, J. , … Daly, M. B. (2014). Preparing individuals to communicate genetic test results to their relatives: report of a randomized control trial. Familial Cancer, 12(3), 537–546. 10.1007/s10689-013-9609-z PMC3706561 23420550

[mgg3940-bib-0021] Newson, A. J. , & Humphries, S. E. (2005). Cascade testing in familial hypercholesterolaemia: How should family members be contacted? European Journal of Human Genetics, 13(4), 401–408. 10.1038/sj.ejhg.5201360 15657617

[mgg3940-bib-0022] Sermijn, E. , Delesie, L. , Deschepper, E. , Pauwels, I. , Bonduelle, M. , Teugels, E. , & De Grève, J. (2016). The impact of an interventional counselling procedure in families with a BRCA1/2 gene mutation: Efficacy and safety. Familial Cancer, 15(2), 155–162. 10.1007/s10689-015-9854-4 26748927 PMC4803813

[mgg3940-bib-0023] Sharaf, R. N. , Myer, P. , Stave, C. D. , Diamond, L. C. , & Ladabaum, U. (2013). Uptake of genetic testing by relatives of lynch syndrome probands: A systematic review. Clinical Gastroenterology and Hepatology, 11(9), 1093–1100. 10.1016/j.cgh.2013.04.044 23669308 PMC12685307

[mgg3940-bib-0024] Smart, A. (2010). Impediments to DNA testing and cascade screening for hypertrophic cardiomyopathy and long QT syndrome: A qualitative study of patient experiences. Journal of Genetic Counseling, 19(6), 630–639. 10.1007/s10897-010-9314-0 20680418

[mgg3940-bib-0025] Sturm, A. C. (2016). Cardiovascular cascade genetic testing: Exploring the role of direct contact and technology. Frontiers in Cardiovascular Medicine, 3, 1–4. 10.3389/fcvm.2016.00011 PMC4835441 27148542

[mgg3940-bib-0026] Suthers, G. K. , Armstrong, J. , McCormack, J. , & Trott, D. (2006). Letting the family know: Balancing ethics and effectiveness when notifying relatives about genetic testing for a familial disorder. Journal of Medical Genetics, 43(8), 665–670. 10.1136/jmg.2005.039172 16371501 PMC2564590

[mgg3940-bib-0027] Van Der Roest, W. P. , Pennings, J. M. , Bakker, M. , Van Den Berg, M. P. , & Van Tintelen, J. P. (2009). Family letters are an effective way to inform relatives about inherited cardiac disease. American Journal of Medical Genetics Part A, 149(3), 357–363. 10.1002/ajmg.a.32672 19213028

[mgg3940-bib-0028] Vavolizza, R. D. , Kalia, I. , Aaron, K. E. , Silverstein, L. B. , Barlevy, D. , Wasserman, D. , … Dolan, S. M. (2015). Disclosing genetic information to family members about inherited cardiac arrhythmias: An obligation or a choice? Journal of Genetic Counseling, 24(4), 608–615. 10.1007/s10897-014-9783-7 25400212 PMC4436086

[mgg3940-bib-0029] Wiseman, M. , Dancyger, C. , & Michie, S. (2010). Communicating genetic risk information within families: A review. Familial Cancer, 9(4), 691–703. 10.1007/s10689-010-9380-3 20852947

