# Supplementary material for: The uptake of family screening in hypertrophic cardiomyopathy and an online video intervention to facilitate family communication
Source: Mol Genet Genomic Med. 2019 Sep 3;7(11):e940. doi: 10.1002/mgg3.940 (PMC6825857; doi:10.1002/mgg3.940)
Supplement: Supplementary file 1 [file MGG3-7-e940-s001.pdf]

HCM SCAMP
SDF2: Overt HCM

BWH MRN: ____________________

Name: ____________________

Visit Date: ____________ **Visit Type:** ☐ Initial evaluation ☐ Follow-up

Attending physician: ☐ Carolyn Ho ☐ Neal Lakdawala ☐ Calum Macrae ☐ Christine Seidman ☐ Travis Hinson

Is this a SCAMP patient? ☐ Yes ☐ No, because: ☐ Infiltrative CMP (ex. amyloid) ☐ Metabolic Cardiomyopathy (ex. Fabry, LAMP2) ☐ One Time Visit ☐ Phenocopy (ex. HTN heart disease) ☐ Other:____________

Genetic Testing Review

Has pt had genetic testing? ☐ Yes → **Skip to First Degree Family Section** ☐ No → **Continue below**

Does the patient meet at least one of the genetic testing criteria: ☐ Concerned for phenocopy ☐ At risk family members

☐ Yes	☐ No
SCAMP recommends: ***Order Genetic Testing***	**Continue to First Degree Family Section**

What is your plan?	☐ Order HCM panel, date:_______<br>☐ Order confirmation testing, date:_______<br>☐ Not order testing
If NOT ordering testing, Reason for deviation:	☐ Obligate carrier<br>☐ Patient not interested<br>☐ Problem with health insurance coverage<br>☐ Other:____________________

***First degree family* member review** *(refer to pedigree)* ➡	# appropriately evaluated ☐<br># at risk ☐	*If did not evaluate first degree family, reason:*	☐ Family not interested ☐ Privacy concerns<br>☐ Concerns about life insurance ☐ Other:____________

Echo Section

Was an Echo recently performed? ☐ Yes, please indicate details below ☐ No

MWT: _________ mm
LVOTO: _________ mmHg

Are there changes on Echo from prior? ☐ Yes, please select changes below: ☐ No Change

(Check all findings as **New** if initial evaluation at BWH.)

☐ Drop in LVEF (>5%) ☐ **New** LVOTO (>30mmHg) ☐ **New** severe LVOTO (>50mmHg) ☐ Other:____________

☐ **Change** in LVOTO (Δ50mmHg) ☐ **New** pulmonary HTN (PASP >40) ☐ Change in MWT:______Δmm, location:______

Did these changes lead to changes in management? ☐ Yes ☐ No

If YES, please indicate changes to management	If NO, does patient meet the below criteria?
☐ SCD risk refinement ☐ Referred for septal reduction<br>☐ Changed medical therapy ☐ Triggered CMR ☐ Other:____________	☐ >2 Echos without change ☐ Stable symptoms for at least 2yrs<br>☐ No family h/o end stage HCM ☐ >3 years since initial dx
SCAMP recommends: ***Order next Echo for 12 mo***	If ALL are met, *SCAMP recommends:* ***Order next Echo for 24 mo***<br>If not, *SCAMP recommends:* ***Order next Echo for 12 mo***
What is your plan? ☐ Order Echo for 12 mo ☐ Order Echo for 24 mo<br>☐ Other: ____________<br>☐ Not order testing	☐ Order Echo for 24 mo ☐ Order Echo for 12 mo<br>☐ Other:____________<br>☐ Not order testing
Reason for deviation: ☐ Previously stable imaging<br>☐ Stable symptoms<br>☐ Other:____________	*Reason for deviation:* ☐ Patient preference<br>☐ Other:____________

HCM SCAMP

SDF2: Overt HCM

CMR Section

Does patient have ICD? ☐ Yes → **Skip to Holter Section** ☐ No → **Continue below**

Has a CMR ever been performed?

☐ Yes, Date:__________	☐ No
SCAMP recommends: ***Do not order CMR***	*SCAMP recommends:* ***Order CMR***
What is your plan? ☐ Not order CMR ☐ Order CMR	What is your plan? ☐ Order CMR ☐ Not order CMR
Reason for deviation: ☐ Patient with no or ≥ 2 SCD risk factors ☐ Patient preference ☐ Other:__________	*Reason for deviation:* ☐ Previously stable imaging ☐ Stable symptoms ☐ Cost ☐ Patient with no or ≥ 2 SCD risk factors ☐ Other:__________

CMR performed in last 6 months? ☐ Yes, Date:________ ☐ No

Were there abnormalities on CMR? ☐ Yes ☐ No

☐ LGE ≥ 15% ☐ Papillary anomaly *

☐ MWT > 30 mm not evident on Echo ☐ Other:__________

Did the abnormalities lead to changes in management? ☐ Yes ☐ No

☐ SCD risk refinement ☐ Changed therapy

☐ Other:____________________

* Papillary anomaly is defined as the presence of direct insertion of the papillary muscle into the mitral apparatus, or papillary displacement encroaching into the outflow tract.

Holter Section

ICD AND known AF and/or anticoagulation? ☐ Yes → **Skip to ETT Section** ☐ No → **Continue Below**

SCAMP recommends: ***Order BNP or NT-BNP***

What is your plan? ☐ Order BNP/NT-BNP ☐ Not order BNP/NT-BNP

Reason for deviation: ☐ Already on anticoagulation ☐ Known AF ☐ Other:____________________

Was a Holter recently performed?

☐ Yes	☐ No
Are there changes on Holter from prior? ☐ Yes ☐ No Change	*SCAMP recommends:* ***Order Holter***
(Check all findings as **New** if initial evaluation at BWH.) ☐ New AF ☐ New NSVT ☐ Other:__________	What is your plan? ☐ Order Holter ☐ Not order Holter ☐ Order 7 day event monitor
Did these changes lead to change in management? ☐ Yes ☐ No	*Reason for deviation:* ☐ ICD with monitoring capabilities ☐ Age >40 ☐ AF ☐ Stable symptoms ☐ Patient preference ☐ Other:__________
☐ Anticoagulation ☐ SCD risk refinement ☐ Adjustment in BB, CCB, or Diso	
**Continue to Event Monitor Section**	**Skip to ETT Section**

NEXT

HCM SCAMP
SDF2: Overt HCM

Event Monitor Section

Are any of these characteristics present?

- ☐ FH of afib
- ☐ Age ≥ 40
- ☐ Diabetes
- ☐ Increased BNP/NT-BNP
- ☐ Palpitations not present on Holter
- ☐ >100 PACs/24hr by Holter
- ☐ Left atrial enlargement by Echo or EKG

- ☐ No characteristics present

SCAMP recommends: ***Order 7 day event monitor***

What is your plan? ☐ Order event monitor ☐ Not ordering event monitor ☐ Order Holter

Reason for deviation: ☐ AF ☐ Patient preference ☐ ICD with monitoring capabilities ☐ On chronic anticoagulation ☐ Other:________________

SCAMP recommends: ***Order Holter for next visit***

What is your plan? ☐ Order Holter ☐ Not ordering Holter ☐ Order 7 day event monitor

Reason for deviation: ☐ Stable symptoms ☐ AF ☐ Age >40 ☐ Patient preference ☐ ICD with monitoring capabilities ☐ Other:______________

ETT Section

Was an ETT performed in the last 6 months? ☐ Yes ☐ No

Are there changes in ETT from prior? ☐ Yes ☐ No Change ☐ Not applicable

(Check all findings as **New** if initial evaluation at BWH.)

- ☐ Exercise capacity declined by ≥ 2 METs
- ☐ Newly abnormal BP response
- ☐ New NSVT
- ☐ Normal BP response (previously abnormal)
- ☐ Other:__________________________

Did these changes lead to change in management? ☐ Yes ☐ No Change ☐ Not applicable

- ☐ SCD risk refinement
- ☐ Adjustment in BB, CCB, or Diso
- ☐ Other:______________________________

Is patient <40 years of age with no ICD?

☐ YES

SCAMP recommends: ***Order ETT in 12 mo***

What is your plan? ☐ Order ETT ☐ Not order ETT

Reason for deviation: ☐ Previously normal ETT ☐ Other:________________

☐ NO

SCAMP recommends: ***Do not order ETT***

What is your plan? ☐ Not order ETT ☐ Order ETT

Reason for deviation: ☐ Labile symptoms ☐ Ongoing SCD risk stratification ☐ Other:______________

NEXT

HCM SCAMP
SDF1: Overt HCM

Please do not complete if patient has an ICD

ICD Recommendations

Are these risk factors present?

- ☐ Secondary prevention
- ☐ Sustained VT (>30 seconds)
- ☐ FH SCD (1 first degree and/or 2 second degree, age <40)
- ☐ End stage-HCM (<LVEF 40%)

☐ YES → SCAMP recommendation: Recommend ICD

☐ NO → Is patient younger than 40 years of age?

☐ Yes → **Are two or more of these risk factors present?**

- ☐ MWT ≥ 3 cm
- ☐ LGE ≥ 15%
- ☐ Syncope (non-vagal, exertional or unexplained within past year)
- ☐ NSVT (≥ 3 beats, ≥ 120 bpm)
- ☐ Hypotensive response to exercise
- ☐ FH SCD not meeting high risk criteria

☐ YES → SCAMP recommendation: Recommend ICD
☐ NO → SCAMP recommendation: Do not recommend ICD

☐ No → **Are two or more of these risk factors present?**

- ☐ MWT ≥ 3 cm
- ☐ LGE ≥ 15%
- ☐ Recent syncope
- ☐ FH SCD not meeting high risk criteria

☐ NO → SCAMP recommendation: Do not recommend ICD
☐ YES → SCAMP recommendation: Recommend ICD

SCAMP recommendation:

Recommend ICD

Your plan

☐ Recommend ICD ☐ Not recommend ICD

If don't recommend ICD, reason for deviation:

- ☐ Advanced age
- ☐ Competing co-morbidity
- ☐ Other: ______________
- ☐ Patient not interested
- ☐ Disagree with SCAMP risk assessment

SCAMP recommendation:

Do not recommend ICD

Your plan

☐ Not recommend ICD ☐ Recommend ICD

If recommend ICD, reason for deviation:

- ☐ Patient Preference
- ☐ Perceived lower risk of complications with subcutaneous ICD
- ☐ Other risk factors considered: ______________
- ☐ High risk lifestyle: ______________
- ☐ Other: ______________
