# Supplementary material for: The uptake of family screening in hypertrophic cardiomyopathy and an online video intervention to facilitate family communication
Source: Mol Genet Genomic Med. 2019 Sep 3;7(11):e940. doi: 10.1002/mgg3.940 (PMC6825857; doi:10.1002/mgg3.940)
Supplement: Supplementary file 2 [file MGG3-7-e940-s002.pdf]

Overt HCM

HCM SCAMP Data Collection Form

BWH MRN: ____________________

Name: ________________________

Visit Date: ____________________

Visit Type: ☐ Initial evaluation ☐ Follow-up

Attending physician: ☐ Carolyn Ho ☐ Neal Lakdawala ☐ Calum Macrae ☐ Christine Seidman

Is this a SCAMP patient? ☐ Yes ☐ No, because: ☐ Infiltrative CMP (ex. amyloid) ☐ Metabolic Cardiomyopathy (ex. Fabry, LAMP2) ☐ One Time Visit ☐ Phenocopy (ex. HTN heart disease) ☐ Other:__________

NYHA Functional Class: ☐ I ☐ II ☐ III ☐ IV

Section 1: Genetic Testing Review

1. Has patient had genetic testing? ☐ No ☐ Yes **(SKIP TO SECTION 2: Family Evaluation)**
2. Is patient an obligate carrier? ☐ No ☐ Yes **(SKIP TO SECTION 2: Family Evaluation)**

Does the patient meet **≥1** of the following criteria?
☐ Concern for phenocopy
☐ At risk family members

☐ Yes ☐ No **(SKIP TO SECTION 2: Family Evaluation)**

SCAMP Recommends: Order genetic testing

What is your plan? ☐ Order genetic testing ☐ Not order genetic testing

If not ordering genetic testing, indicate reason(s) for deviation:

☐ Patient not interested ☐ Problem with health insurance coverage ☐ Other:__________

Section 2: Family Member Evaluation and Patient Engagement

***SCAMP Recommends*:**
Evaluate all at risk first degree family members

Please indicate the fraction of family members evaluated:
Appropriately Evaluated ☐
At Risk ☐

Appropriately evaluated =
Age <25: normal echo in last 2 yrs
Age 25-60: normal echo in last 3 yrs
Age >60: normal echo in last 5 yrs

At Risk = living parents, siblings, and children (1st degree family members) presumed unaffected by HCM, who are >9 yrs old

# of family members newly evaluated since last visit: ☐

☐ <100% evaluated ☐ 100% evaluated

Reasons for not evaluating first degree family members:

☐ New Diagnosis ☐ Patient Unaware ☐ Patient aware, but has not communicated w/ family
☐ Family aware, but not interested ☐ Other:____________________

***SCAMP Recommends*:** Send Vidscrip to patient

What is your plan? ☐ Sending Vidscrip ☐ Not sending Vidscrip

If sending Vidscrip, indicate your plan:
☐ Provide link to website*
☐ Provide QR code for a phone scanner**
☐ Provide Vidscrip postcard
☐ Other:__________

If not sending Vidscrip, indicate reason for deviation:
____

Of at risk family members, are **>75% OR ≥3 1st degree family members** NOT evaluated?

☐ Yes ☐ No

Skip to next page 'Section 3: Echocardiogram'

***SCAMP Recommends*:** Arrange formal revisit with genetic counselor

What is your plan? ☐ In clinic revisit ☐ Telephone revisit ☐ No revisit

If not scheduling a revisit, indicate reason(s) for deviation:
☐ Family aware, but not interested ☐ New Diagnosis ☐ Other:__________

Note: Vidscrip website works on *Chrome* and *Safari* browsers
*http://www.vidscrip.com/bwhcardiovasculargenetics/
**QR code:

Section 3: Echocardiogram Results

1. Was an echo recently performed? ☐ Yes, date: ____________ ☐ No **(SKIP TO SECTION 4: Echo Utilization)**
   (within 9 months)

   Was echo performed at BWH?

   ☐ YES ☐ No

   Please indicate findings:

   LVOTO:__________mmHg (baseline)
   LVOTO:__________mmHg (provoked)
   MWT:____________mm
   LA Size (AP diameter):____________mm
   PASP:__________mmHg [ ]unavailable

2. Are there changes on echo from prior? ☐ Yes, please indicate: ☐ No ☐ N/A (patient's 1st echo)
   - ☐ Drop in LVEF (>5%)
   - ☐ Change in MWT (≥2mm) location:______
   - ☐ New LVOTO (>30 mmHg)
   - ☐ New severe LVOTO (>50mmHg)
   - ☐ Change in LVOTO (>50 mmHg)
   - ☐ New Pulmonary HTN (PASP >40)
   - ☐ Other:____________________

3. Did this echo result in changes in management? ☐ Yes, please indicate: ☐ No ☐ N/A (patient's 1st echo)
   - ☐ SCD risk refinement
   - ☐ Changed medical therapy
   - ☐ Referred for septal reduction
   - ☐ Triggered CMR
   - ☐ Other:____________________

Section 4: Echocardiogram Utilization

Does the patient meet **≥3** of the following low risk criteria?

- ☐ Stable symptoms for ≥2 years
- ☐ Previously stable imaging
- ☐ >3 years since initial dx
- ☐ No family h/o end stage HCM

☐ Yes ☐ No

If Yes:

SCAMP Recommends:
Order echo 24 months from prior

What is your plan? ☐ Order 24 mth echo ☐ Order 12 mth echo ☐ Other: ________

Reasons for deviation:

- ☐ Patient preference
- ☐ Other: ________

If No:

SCAMP Recommends:
Order echo 12 months from prior

What is your plan? ☐ Order 12 mth echo ☐ Order 24 mth echo ☐ Other: ________

Reasons for deviation:

- ☐ Previously stable imaging
- ☐ Stable symptoms
- ☐ Other: ________

Section 5: Monitoring

Was monitoring performed in the last 6 months?

☐ Yes ☐ No

Please indicate type of monitoring:

- ☐ Holter
- ☐ 7 day event monitor
- ☐ 14 day event monitor
- ☐ Other: ______________

Does patient have a dual chamber ICD, known AF and/or already on anticoagulation?

☐ Yes ☐ No

Does patient have ANY of these AF risk factors?

- ☐ FH of AF
- ☐ NT-BNP > ULN of assay (>449 pg/mL)
- ☐ >100 PAC/24 hr on Holter
- ☐ Age ≥40
- ☐ Palpitations not present during monitoring
- ☐ LAE (LA >40mm by echo OR ≥mild LAE by CMR)

☐ Yes ☐ No

SCAMP Recommends:
Do Not Order Monitoring

SCAMP Recommends:
1 - 2 AF Risk Factors - Order 7 day event monitor
3 AF Risk Factors - Order 14day event monitor
≥ 4 AF Risk Factors - Order 14day event monitor and ECG check

SCAMP Recommends:
Order Holter

What is your plan?

☐ Not ordering monitoring ☐ Ordering 7 day event monitor ☐ Ordering 14 day event monitor ☐ Ordering Holter

☐ ECG Check ☐ Other:__________

If applicable, please indicate reason for deviation from SCAMP:

- ☐ ICD with monitoring capabilities
- ☐ Known AF
- ☐ Stable symptoms
- ☐ Patient does not have smart phone
- ☐ On chronic anticoagulation
- ☐ Patient preference
- ☐ Other: ______________________

Section 6: ICD Placement

Does patient have an ICD?

- ☐ Yes **(FORM COMPLETE)**
- ☐ No

Are these risk factors present?

- ☐ Secondary prevention
- ☐ Sustained VT (>30 seconds)
- ☐ FH SCD (1 first degree and/or 2 second degree, age <40)
- ☐ End stage - HCM (LVEF <50%)

☐ Yes ☐ No

Is patient <40 years old?

☐ Yes ☐ No

(Yes) Are ≥ 2 of these risk factors present?

- ☐ MWT ≥ 3 cm
- ☐ LGE ≥ 15%
- ☐ Syncope (non-vagal, exertional or unexplained within past year
- ☐ NSVT (≥3 beats, ≥120 bpm)
- ☐ Hypotensive response to exercise
- ☐ FH SCD not meeting high risk criteria

☐ Yes ☐ No

(No) Are ≥ 2 of these risk factors present?

- ☐ MWT ≥ 3 cm
- ☐ LGE ≥15%
- ☐ Recent syncope
- ☐ FH SCD not meeting high risk criteria

☐ Yes ☐ No

SCAMP Recommends:
Recommend ICD

What is your plan? ☐ Recommend ICD ☐ Do Not Recommend ICD

Reasons for deviation:

- ☐ Advanced Age
- ☐ Competing co-morbidity
- ☐ Patient not Interested
- ☐ Disagree with SCAMP risk assessment
- ☐ Other:__________

SCAMP Recommends:
Do Not Recommend ICD

What is your plan? ☐ Do Not Recommend ☐ Recommend ICD

Reasons for deviation:

- ☐ Patient Preference
- ☐ Perceived lower risk of complication with subcutaneous ICD
- ☐ Other risk factors considered:__________
- ☐ High risk lifestyle: __________
- ☐ Other:__________
