# Supplementary material for: The uptake of family screening in hypertrophic cardiomyopathy and an online video intervention to facilitate family communication
Source: Mol Genet Genomic Med. 2019 Sep 3;7(11):e940. doi: 10.1002/mgg3.940 (PMC6825857; doi:10.1002/mgg3.940)
Supplement: Supplementary file 3 [file MGG3-7-e940-s003.pdf]

Supplementary Material

https://app.vidscrip.com/user/5726caf8a254a5897c3c384a
