# Supplementary material for: The uptake of family screening in hypertrophic cardiomyopathy and an online video intervention to facilitate family communication
Source: Mol Genet Genomic Med. 2019 Sep 3;7(11):e940. doi: 10.1002/mgg3.940 (PMC6825857; doi:10.1002/mgg3.940)
Supplement: Supplementary file 4 [file MGG3-7-e940-s004.pdf]

VidScrip Survey

Please complete the survey below.

Thank you!

Identifier ______________________________

Number of relatives that have been screened ______________________________

Number of relatives that should have be screened ______________________________

We are asking you to participate in a research study called "Assessing familial communication of genetic risk and the utility of interventions to facilitate the dissemination of familial risk information in inherited cardiovascular disease". The purpose of this study is to help us better understand how patients communicate with their relatives about genetic risk. This survey will take approximately 15 minutes to complete. You will not be paid for participating in this survey.

Completing this survey is voluntary. If you choose not to participate, there will be no consequences or changes in your care at Brigham and Women's Hospital. Only members of the study team will have the link between your name and your survey responses. The risk of allowing us to record your name with your answers is a loss of confidentially. We will take reasonable steps to respect the confidentiality of your information. Would you be willing to answer questions about your health and medical history to find out if you might qualify for the study? Some of the questions may make you feel uncomfortable. You may stop at any time.

The Principal Investigator for this study is Dr. Neal Lakdawala. If you have any questions regarding the survey, please contact the study coordinator, Stephanie Harris at 617-525-7168. If you'd like to speak to someone not involved in this research about your rights as a research subject, or any concerns or complaints you may have about the research, contact the Partners Human Research Committee at 857-282-1900.

By clicking "yes", you agree to participate in this study and will be directed to the survey.

- ◯ Yes
- ◯ No

Did you watch the HCM VidScrip provided by Brigham and Women's Cardiovascular Genetics Clinic?

- ◯ Yes
- ◯ No
- ◯ I don't remember

Did the VidScrip increase your understanding of the risk of HCM to family members?

- ◯ Not at all
- ◯ A little
- ◯ A lot
- ◯ I did not learn any new information, but it was a helpful review

Did the VidScrip increase your understanding of screening recommendations for family members?	◯ Not at all<br>◯ A little<br>◯ A lot<br>◯ I did not learn any new information, but it was a helpful review
Did you share the VidScrip with any of your relatives?	◯ Yes<br>◯ No<br>◯ I don't remember
Did you send the VidScrip to relatives with whom you have already shared your diagnosis?	◯ Yes<br>◯ No
Did you send the VidScrip to relatives with whom you have not already shared your with diagnosis?	◯ Yes<br>◯ No
Have any of your relatives decided to get evaluated after watching the VidScrip?	◯ Yes<br>◯ No<br>◯ I don't know
How many of your relatives have been evaluated after watching the VidScrip	__________________________________<br>(Please provide numerical value)

Please rate how important each of the following reasons are for why you sent the VidScrip to your family members.

	Not Important (1)	Of Little Importance (2)	Moderately Important (3)	Important (4)	Very Important (5)
Easy to send online	◯	◯	◯	◯	◯
My family member prefers email	◯	◯	◯	◯	◯
It was easier to send online because I do not usually speak to this family member in person	◯	◯	◯	◯	◯
The VidScrip contains all the information I would tell a family member	◯	◯	◯	◯	◯
The VidScrip would ensure I am telling my family member the correct information	◯	◯	◯	◯	◯
Fast way to share information	◯	◯	◯	◯	◯
Easy to share information with many people at once	◯	◯	◯	◯	◯

Please rate how important each of the following reasons are for why you DID NOT send the VidScrip to your family members

	N/A	Not Important (1)	Of Little Importance (2)	Moderately Important (3)	Important (4)	Very Important (5)
Technical problems	○	○	○	○	○	○
Did not know the family member's email address	○	○	○	○	○	○
The family member does not have internet access	○	○	○	○	○	○
The VidScrip was too much information	○	○	○	○	○	○
The VidScrip was not enough information	○	○	○	○	○	○
The VidScrip was too difficult to understand	○	○	○	○	○	○
Privacy concerns about sharing over the internet	○	○	○	○	○	○
