# Supplementary material for: The uptake of family screening in hypertrophic cardiomyopathy and an online video intervention to facilitate family communication
Source: Mol Genet Genomic Med. 2019 Sep 3;7(11):e940. doi: 10.1002/mgg3.940 (PMC6825857; doi:10.1002/mgg3.940)
Supplement: Supplementary file 5 [file MGG3-7-e940-s005.pdf]

Family Communication Survey

Please complete the survey below.

Thank you!

Identifier ______________________________

We are asking you to participate in a research study called "Assessing familial communication of genetic risk and the utility of interventions to facilitate the dissemination of familial risk information in inherited cardiovascular disease". The purpose of this study is to help us better understand how patients communicate with their relatives about genetic risk. This survey will take approximately 15 minutes to complete. You will not be paid for participating in this survey.

Completing this survey is voluntary. If you choose not to participate, there will be no consequences or changes in your care at Brigham and Women's Hospital. Only members of the study team will have the link between your name and your survey responses. The risk of allowing us to record your name with your answers is a loss of confidentially. We will take reasonable steps to respect the confidentiality of your information. Would you be willing to answer questions about your health and medical history to find out if you might qualify for the study? Some of the questions may make you feel uncomfortable. You may stop at any time.

The Principal Investigator for this study is Dr. Neal Lakdawala. If you have any questions regarding the survey, please contact the study coordinator, Stephanie Harris at 617-525-7168. If you'd like to speak to someone not involved in this research about your rights as a research subject, or any concerns or complaints you may have about the research, contact the Partners Human Research Committee at 857-282-1900.

By clicking "yes", you agree to participate in this study and will be directed to the survey.

○ Yes
○ No

Have you participated in the VidScrip Survey?

○ Yes
○ No

Family Evaluation

How many living, biological parents do you have?

____
(Please provide a numerical value)

How many have been evaluated for your condition?

____
(Please provide a numerical value)

How many living, biological siblings do you have?

____
(Please provide a numerical value)

How many have been evaluated for your condition?

____
(Please provide a numerical value)

How many living, biological children do you have?

____
(Please provide a numerical value)

How many of your children have been evaluated for your condition?

____
(Please provide a numerical value)

Communication Practices

Have you shared your diagnosis with any of the following people? Check all that apply.

- ☐ All living, biological siblings
- ☐ Some living, biological siblings
- ☐ All living, biological children
- ☐ Some living, biological children
- ☐ Biological mother (if alive)
- ☐ Biological father (if alive)
- ☐ Living, biological extended family (aunt/uncle, nephew/niece)
- ☐ I have not shared my diagnosis with any family members, but I plan to share this information
- ☐ I have not shared my diagnosis and I do not plan to share this information
- ☐ I do not have any living, biological relatives

What topics were discussed with family members? Check all that apply.

- ☐ Their risk for the condition
- ☐ The recommendation for them to get evaluated for the condition
- ☐ Feelings about the diagnosis
- ☐ Genetic testing
- ☐ Other

What other topics did you discuss?

____

Please rate how important each of the following reasons are for why you shared your diagnosis with your family members.

	Not Important (1)	Of Little Importance (2)	Moderately Important (3)	Important (4)	Very Important (5)
I wanted them to be informed of their own risk	◯	◯	◯	◯	◯
I wanted to encourage them to get evaluated	◯	◯	◯	◯	◯
I wanted their emotional support	◯	◯	◯	◯	◯
I wanted to get advice regarding my medical care	◯	◯	◯	◯	◯
My doctor or genetic counselor told me to tell them	◯	◯	◯	◯	◯

Which method of communication did you use to share your diagnosis with family members? Check all that apply.

- ☐ In-person
- ☐ Over the phone
- ☐ Letter
- ☐ Email
- ☐ Social media
- ☐ Text message
- ☐ Other

What other methods did you use?

____

Please rate how important each of the following reasons are for why you DID NOT share your diagnosis with your family members.

	N/A, I shared my diagnosis with all my relatives	Not Important (1)	Of Little Importance (2)	Moderately Important (3)	Important (4)	Very Important (5)
I did not want to worry them	◯	◯	◯	◯	◯	◯
We do not have a close relationship	◯	◯	◯	◯	◯	◯
We do not live close to each other	◯	◯	◯	◯	◯	◯
I did not think they would understand the information	◯	◯	◯	◯	◯	◯
I am worried about confidentiality	◯	◯	◯	◯	◯	◯
I did not want it to negatively impact our relationship	◯	◯	◯	◯	◯	◯
I feel guilty about my result	◯	◯	◯	◯	◯	◯
They would not care	◯	◯	◯	◯	◯	◯
They are not at risk	◯	◯	◯	◯	◯	◯

I did not know what to say	○	○	○	○	○	○
I am having difficulty coping	○	○	○	○	○	○
My relatives do not have the financial resources for evaluations and/or genetic testing	○	○	○	○	○	○
My family members already know they are at risk	○	○	○	○	○	○

Communication Preferences

Which of the following would you find helpful when sharing your diagnosis with family members? Check all that apply.

- ☐ A short brochure about your condition
- ☐ A detailed packet of information about your condition
- ☐ An educational video about your condition
- ☐ A personalized letter about your condition from your healthcare provider
- ☐ None of these
- ☐ Other

What else would you find helpful?

____

In general, how is medical information shared within your family? Please choose the most appropriate option.

	Never	Sometimes	Often
In-person	○	○	○
Over the phone	○	○	○
Letter	○	○	○
Email	○	○	○
Social media	○	○	○
Text message	○	○	○

Are you interested in receiving additional guidance from your healthcare providers to help you communicate your diagnosis to your family members?

- ○ Yes
- ○ No

Hypothetically, with your permission, would you want your healthcare providers to contact your family members directly to share information about their risk for the condition and the recommended evaluations?

- ○ Yes, all of my at-risk family members
- ○ Some of my at-risk family members
- ○ Yes, all of my at-risk family members, but only after I had the chance to speak with them
- ○ Yes, some of my at-risk family members, but only after I had the chance to speak with them
- ○ No, none of my at-risk family members
- ○ Unsure

Do you have a family member who takes the lead in communicating medical matters within your family?

- ○ Yes
- ○ No

What is this person's relationship to you?

____
(Please note, we will not contact this person)

What is this person's approximate age?	___________________________________<br>(Please note, we will not contact this person)
What is this person's gender?	◯ Male<br>◯ Female<br>(Please note, we will not contact this person)
What is this person's preferred method of communication for sharing medical information?	___________________________________<br>(Please note, we will not contact this person)
Would you share our contact information below with that family member so we can see if they are interested in participating in future research studies about family communication?<br><br>Contact information:<br>Stephanie Harris<br>Genetic Counselor<br>Cardiovascular Genetics Center<br>Telephone: 617.525.7168	◯ Yes<br>◯ No<br>(Please note, we will not initiate contact with this person nor discuss your medical history, but we will speak with them about future family communication research studies if they contact us.)
Is there something more you wish your healthcare providers could do to help you share your diagnosis with your family members?	___________________________________

Thank you for participating in this survey!
